# N-acetylcysteine prevents bladder tissue fibrosis in a lipopolysaccharide-induced cystitis rat model

**DOI:** 10.1038/s41598-019-44631-3

**Published:** 2019-05-31

**Authors:** Chae-Min Ryu, Jung Hyun Shin, Hwan Yeul Yu, Hyein Ju, Sujin Kim, Jisun Lim, Jinbeom Heo, Seungun Lee, Dong-Myung Shin, Myung-Soo Choo

**Affiliations:** 1 Department of Urology, University of Ulsan College of Medicine, Seoul, Korea; 2 Department of Biomedical Sciences, University of Ulsan College of Medicine, Seoul, Korea; 3 Department of Physiology, Asan Medical Center, University of Ulsan College of Medicine, Seoul, Korea

**Keywords:** Bladder, Mesenchymal stem cells

## Abstract

Therapeutic options for non-Hunner type interstitial cystitis (IC), which is histologically characterized by fibrosis and mast cell infiltration, are limited. We developed a rat model that replicates chronic inflammation and fibrosis and evaluated the therapeutic effect of N-acetylcysteine (NAC), a well-known anti-fibrotic agent, on the model. Intravesical instillation of lipopolysaccharide (LPS, 750 μg) after protamine sulfate (10 mg) was conducted twice per week for five consecutive weeks. One week after final instillation, 200 mg/kg NAC (n = 10, IC + NAC group) or phosphate-buffered saline (n = 10, IC group) was daily injected intraperitoneally once daily for 5 days. LPS instillation induced bladder fibrosis, mast cell infiltration, and apoptotic tissue damage. Functionally, LPS insult led to irregular micturition, decreased inter-contraction intervals, and decreased micturition volume. NAC significantly improved most of the voiding parameters and reversed histological damages including fibrosis. NAC inhibited the induction and nuclear localization of phospho-Smad2 protein in bladder tissues and the upregulation of genes related to fibrosis, such as *Tgfb2*, *Tgfb3*, *Smad2*, *Smad3*, *Cxcl10*, and *Card10*. This is the first study to demonstrate the beneficial effects on NAC in restoring voiding function, relieving tissue fibrosis and related bladder injuries, in the LPS-induced cystitis rat model.

## Introduction

Interstitial cystitis/bladder pain syndrome (IC/BPS) is a chronic pelvic condition that is usually associated with pain, increased urinary frequency, nocturia, and urgency without evidence of urinary tract infection and other identifiable causes^1^. Clinically, IC/BPS could be subcategorized as Hunner type (IC) and non-Hunner type (BPS) depending on the presence of Hunner lesions on cystoscopy^2^. There is no consensus on the exact pathophysiology of IC/BPS; however, various mechanisms, including mast cell infiltration^3–5^, glycosaminoglycan layer defect^6^, and autoimmune upregulation^7–9^, have been suggested. In addition, to increase the understanding of the disease, several animal models have been developed to reproduce the bladder-related features of IC/BPS through the application of intravesical or systemic toxic stimuli^10,11^.

In bladder tissue from patients with IC/BPS, non-Hunner-type IC has been shown to cause fibrosis and mast cell infiltration, whereas Hunner-type IC caused urothelium denudation and inflammation^12^. Fibrosis, an excess accumulation of the extracellular matrix, is a common pathological feature of most chronic inflammatory diseases, which can eventually lead to organ dysfunction^13^. In urinary bladder, fibrosis can result in decreased compliance and capacity, leading to dysfunctional voiding and possible upper tract damage^14^. Fibrosis of bladder might result from substance-induced cystitis such as ketamine or cyclophosphamide^15^, chronic radiation cystitis^16^, and in some cases of IC/BPS.

Treatment modalities for bladder fibrosis are limited, and establishment of an animal model representing chronic inflammation and accompanied fibrotic change in bladder is important to develop a novel therapeutic strategy. To replicate the chronic injury of IC, we carried out long-term intravesical instillation of lipopolysaccharide (LPS) once weekly for 5 weeks in our previous study^17^. This previous model showed prominent urothelial denudation and inflammation like Hunner-type IC; however, meaningful fibrotic changes were not induced.

N-acetylcysteine (NAC), a precursor of glutathione, is an antioxidant that directly scavenges oxygen free radicals and alters the structure of transforming growth factor-β (TGF-β) to attenuate pro-fibrotic activities^18^. It is clinically used as a mucolytic drug, an agent for preventing lung fibrosis in idiopathic pulmonary fibrosis, and a hepatoprotective agent^19^. Recently, NAC has been found to be effective against neutrophilic airway inflammation in patients with cystic fibrosis^20^ and as a neuroprotective agent in a brain injury animal model^21^.

Previously, we reported that ketamine, (which is a noncompetitive N-methyl-D-aspartic acid receptor antagonist), induces fibrotic changes in the bladder of a rat model in a dose-dependent manner^22^. In addition, intraperitoneal injection of NAC significantly alleviated fibrosis of the bladder and improved voiding dysfunction in the ketamine-induced cystitis rat model^23,24^. In the present study, our primary aim was to develop a rat model that presents prominent fibrosis and mast cell infiltration in the bladder. Further, we aimed to demonstrate the therapeutic effects of NAC in the LPS-induced cystitis rat model.

## Results

### Induction of fibrotic damage in the IC rat model through double instillation of LPS

**Figure 1:**
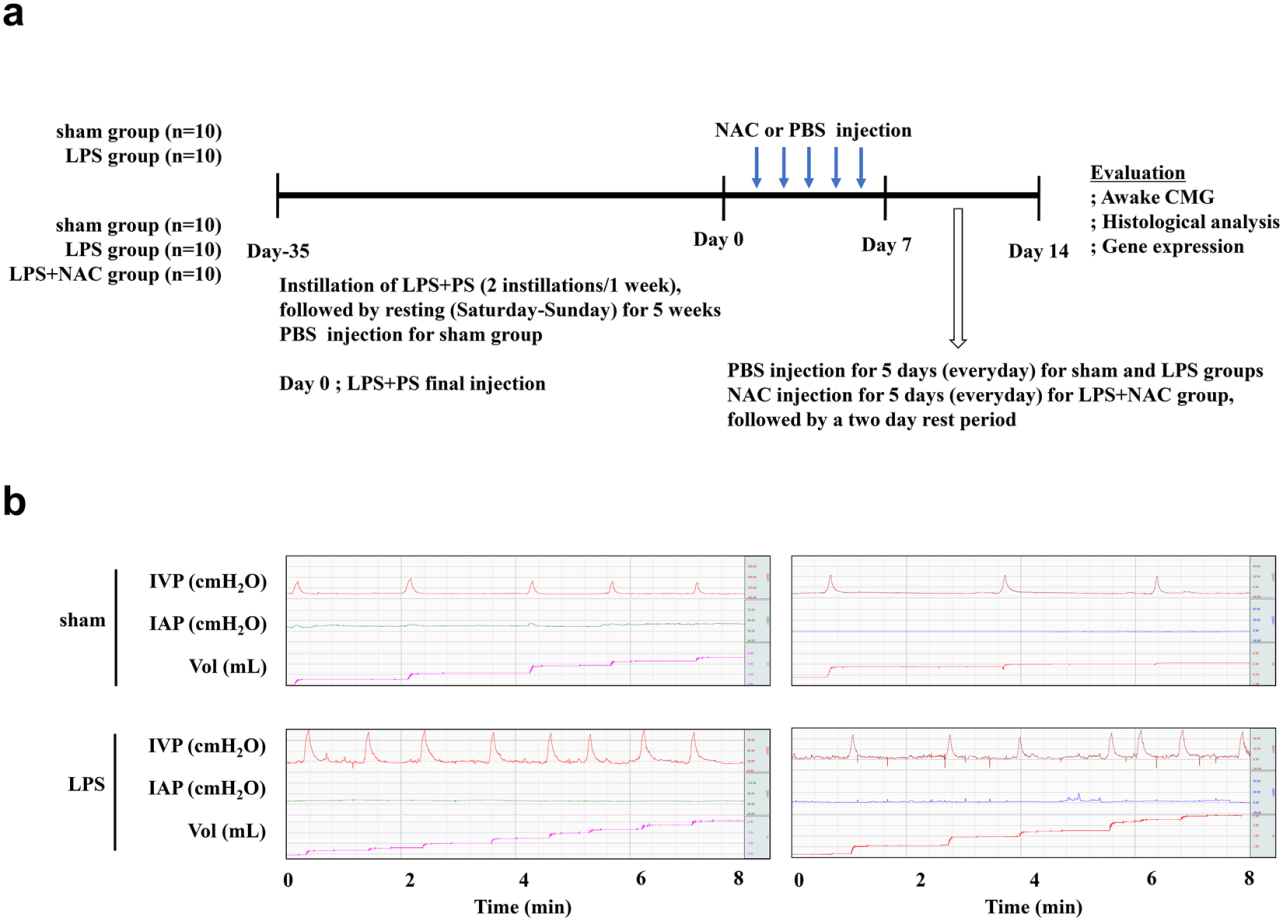
Phosphate-buffered saline injection ameliorated the bladder voiding function in the lipopolysaccharide-induced cystitis rat model. (**a**) Schematic diagram of the study design (**b**) awake cystometry. Representative awake cystometry results of the indicated groups are shown. IVP, intravesical pressure; IAP, intra-abdominal pressure; Sham, sham operated.

In our previous IC rat model, instillation of LPS and PS induced long-lasting and chronic urothelial injury that accurately mimics the chronic and inflammatory nature of IC^17^. However, this model did not reflect the fibrotic response that is well correlated with increased urinary frequency and decreased bladder capacity in patients with IC^12^. To induce more severe fibrosis, we increased the number of instillations of LPS and PS into the bladder to 2 times a week for 5 weeks (Fig. 1a and Supplementary Fig. 1). First, we performed awake filling cystometry, which allows long-term evaluation of bladder voiding function in free-moving animals, thus enabling precise investigation of the symptoms of IC. As shown in (Fig. 1b), compared with the sham group, rats with LPS-induced IC (IC group) exhibited irregular voiding pattern and decreased MI (89.89 ± 1.03 vs. 19.89 ± 0.32 s, respectively; p < 0.05), BC (0.54 ± 0.03 vs. 0.13 ± 0.13 mL, respectively; p < 0.05), MV (0.24 ± 0.01 vs. 0.11 ± 0.0 mL, respectively; p < 0.01), and RV (0.35 ± 0.01 vs. 0.01 ± 0.0 mL, respectively; p < 0.05).

**Figure 2:**
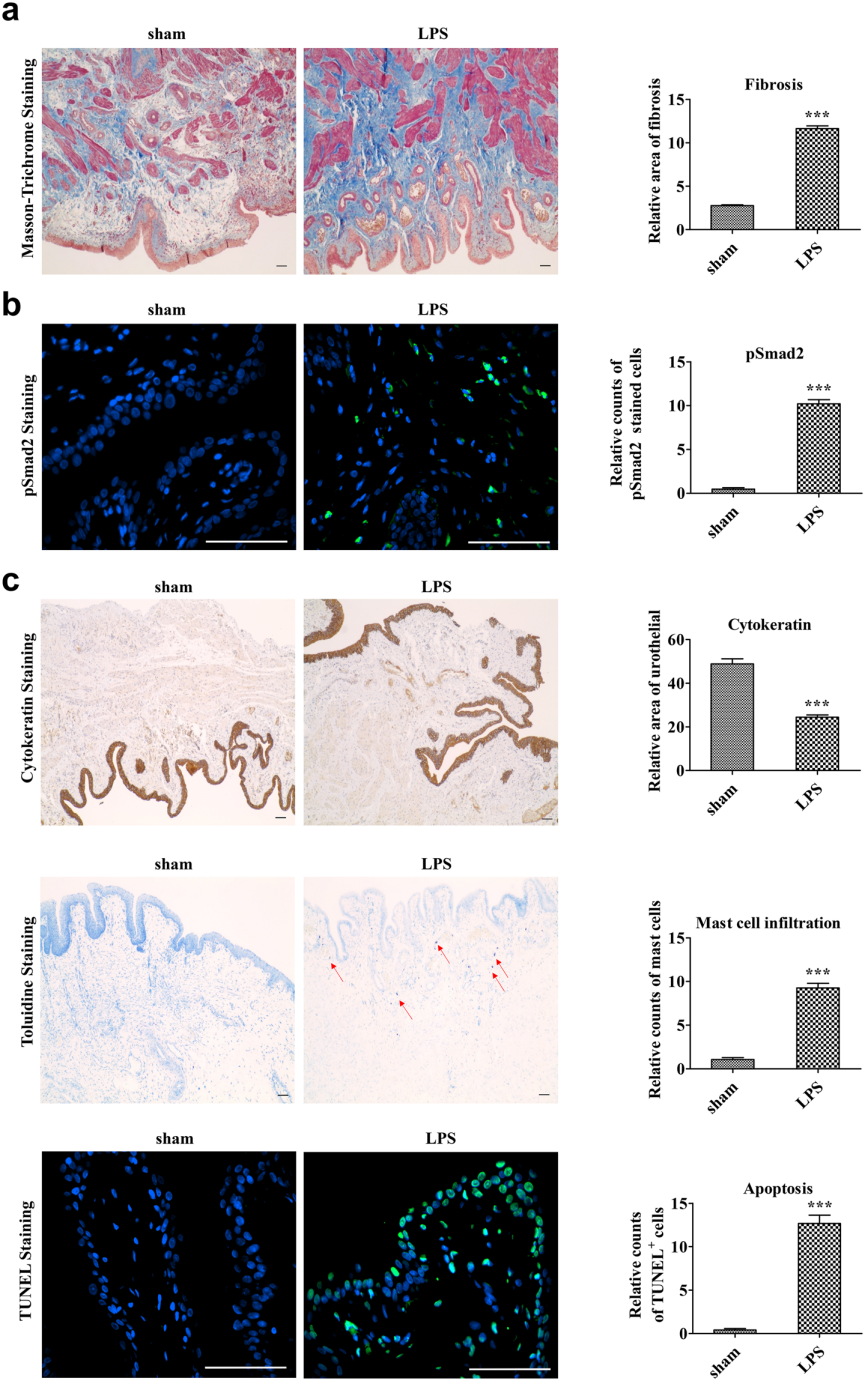
Histological analysis of lipopolysaccharide-induced bladder injuries. (**a**) Masson’s trichrome staining (magnification × 100, scale bar = 100 μm), (**b**) phosphorylated Smad2 protein (green) staining (magnification × 400), (**c**) cytokeratin immunostaining (magnification × 40, scale bar = 100 μm), toluidine blue staining (magnification × 100, scale bar = 100 μm), and TUNEL staining (magnification × 400, scale bar = 100 μm) of bladder tissues of lipopolysaccharide-induced interstitial cystitis (LPS) rats 1 week after phosphate-buffered saline injection. Arrows indicate infiltrated mast cells or apoptotic cells. Sham, sham operated. Quantitative data of each staining are presented on the right side of the indicated representative pictures. Data were normalized to the sham group (n = 15). Data are presented as mean ± standard error of the mean. **p < 0.01, ***p < 0.001 compared with the LPS group with Bonferroni post-test.

In line with the impairment in bladder voiding functions, the double instillation of LPS and PS induced tissue fibrosis, as shown by the results of Masson’s trichrome staining (Fig. 2a). We next examined the levels of p-Smad2 protein, a surrogate marker of activated TGF-β signaling that plays a key role in tissue fibrosis^23^. Immunohistochemical analysis detected nuclear staining of p-Smad2 proteins in the bladders of the IC group but not in the sham group (Fig. 2b). Similar to our previous study with a single dose of LPS installation^17^, the bladders of the LPS group exhibited marked decreases in cytokeratin-stained urothelium, increased toluidine blue-stained mast cells’ infiltration, and a higher number of TUNEL-stained apoptotic cells (Fig. 2c), in comparison with the sham group. Taken together, these results indicate that the model of IC induced with the double-frequency LPS regimen replicates the fibrotic damage concomitant with bladder voiding and histological features characteristic to patients with IC.

### Evaluation of the *in vivo* therapeutic potency of NAC for treating IC/BPS

**Figure 3:**
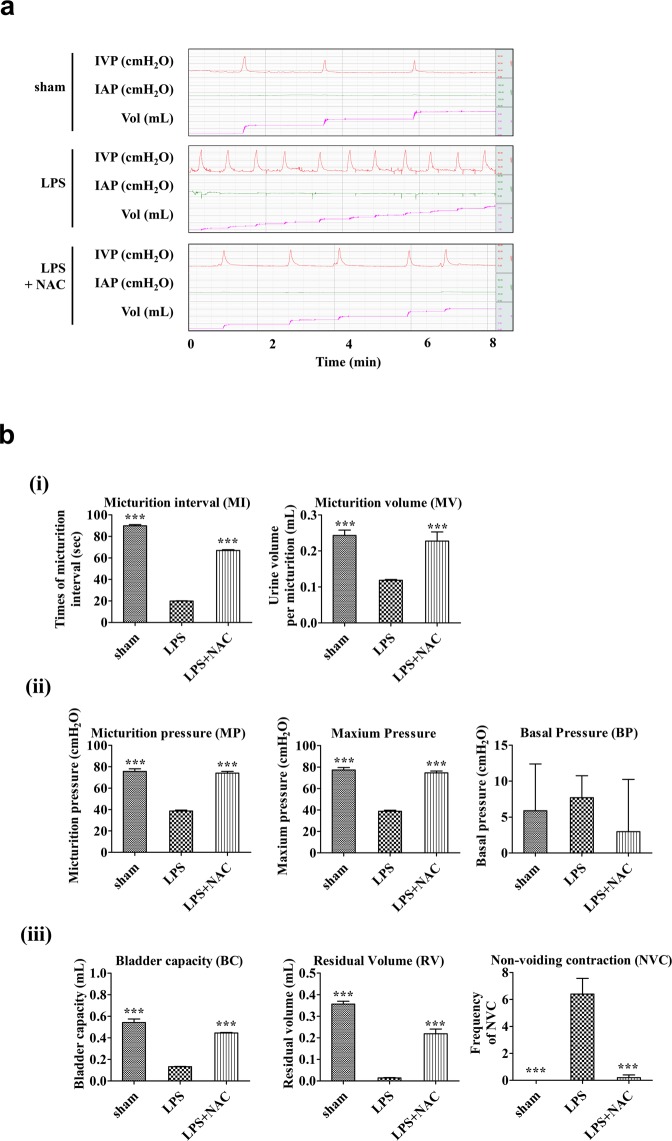
N-acetylcysteine (NAC) injection ameliorated the bladder voiding function in the lipopolysaccharide-induced cystitis (LPS) rat model. (**a**) Awake cystometry. (**b**) Representative awake cystometry results and quantitative bladder voiding parameters at 1 week after NAC injection. Data are presented as mean ± standard error of the mean (from 10 independent animals in each group). *p < 0.05, **p < 0.01, ***p < 0.001 compared with the LPS group with Bonferroni post-test. IVP, intravesical pressure; IAP, intra-abdominal pressure; Sham, sham operated.

We next examined the *in vivo* efficacy of NAC, an anti-fibrotic small molecule, on the IC animal model. To address this issue, after double instillation of LPS for 5 weeks, NAC at 200 mg/kg was daily administered for 5 days through the intraperitoneal route, and then the therapeutic outcomes were evaluated using a bladder voiding function test as well as histological and gene expression examinations of bladder tissue. As shown in Fig. 3a, treatment of NAC significantly improved the voiding dysfunction observed in the bladders of the LPS group. The decreases in MI, MV, MP, maximum pressure, BC, and RV observed in IC group rats were ameliorated through daily intraperitoneal injection of NAC (Fig. 3b). In particular, NAC therapy remarkably prevented the increased frequency of contraction during non-voiding periods, which usually represents the symptom of urinary urgency in a clinical setting (Fig. 3c).

### NAC therapy prevented tissue fibrosis and inflammation in LPS-treated bladders

**Figure 4:**
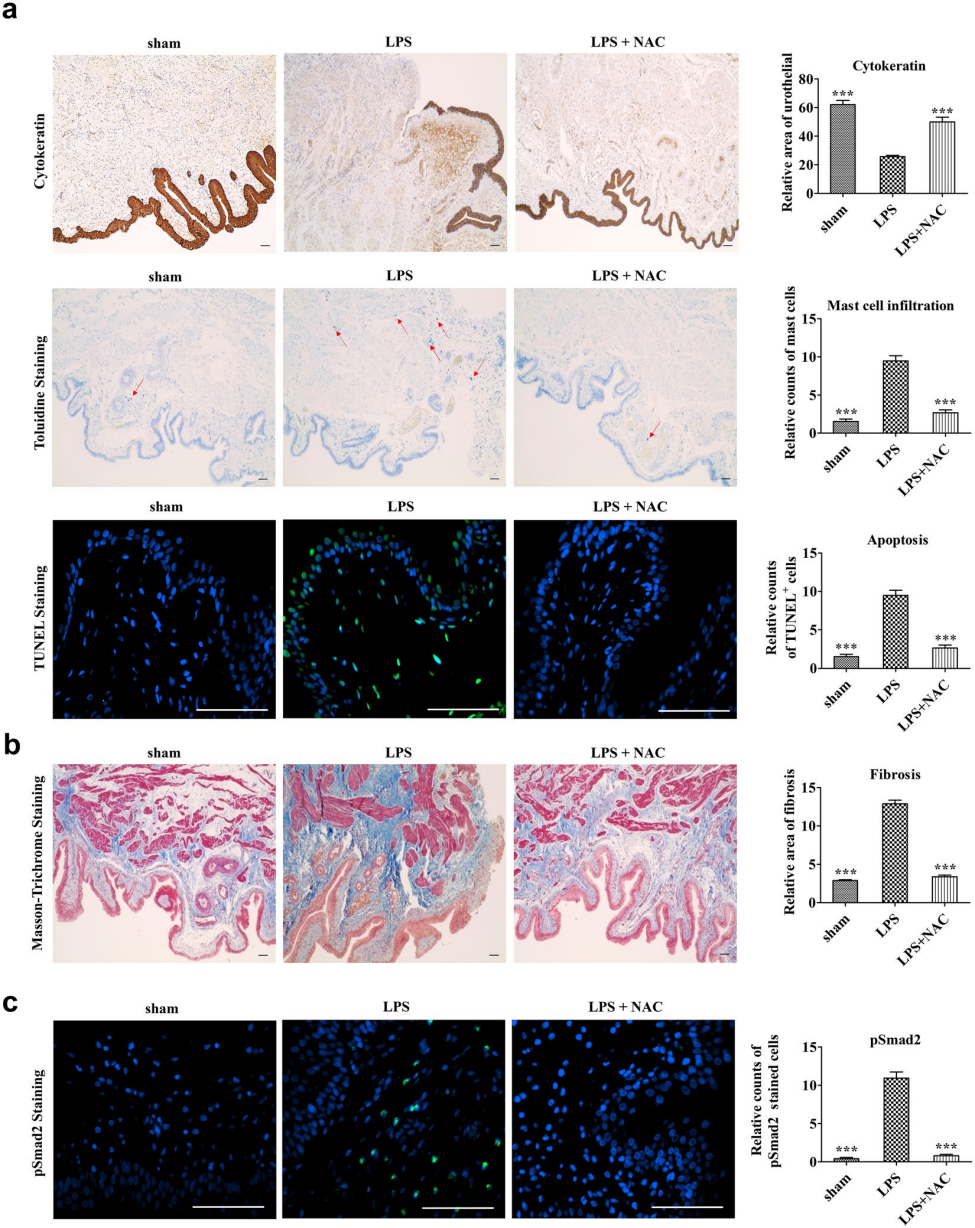
Histological analysis of the effect of N-acetylcysteine (NAC) pharmacologic therapy on the bladder of lipopolysaccharide-induced cystitis (LPS) rat model. Histological analysis of fibrosis, TGF-β activation, mast cell infiltration, and apoptosis in the bladder tissues of the indicated groups. Fluorescent immunohistochemical detection of phosphorylated Smad2 protein (green) in the indicated bladder tissues (magnification × 400). Nuclei were stained with 4′,6-diamino-2-phenylindole (blue). Quantitative data of each staining are presented on the right side of the indicated representative pictures. All quantitative data were normalized to those of the sham group and are presented as dot plot with mean ± standard error of the mean (n = 15). *p < 0.05, **p < 0.01, ***p < 0.001 compared with the LPS with the Bonferroni post-test.

**Figure 5:**
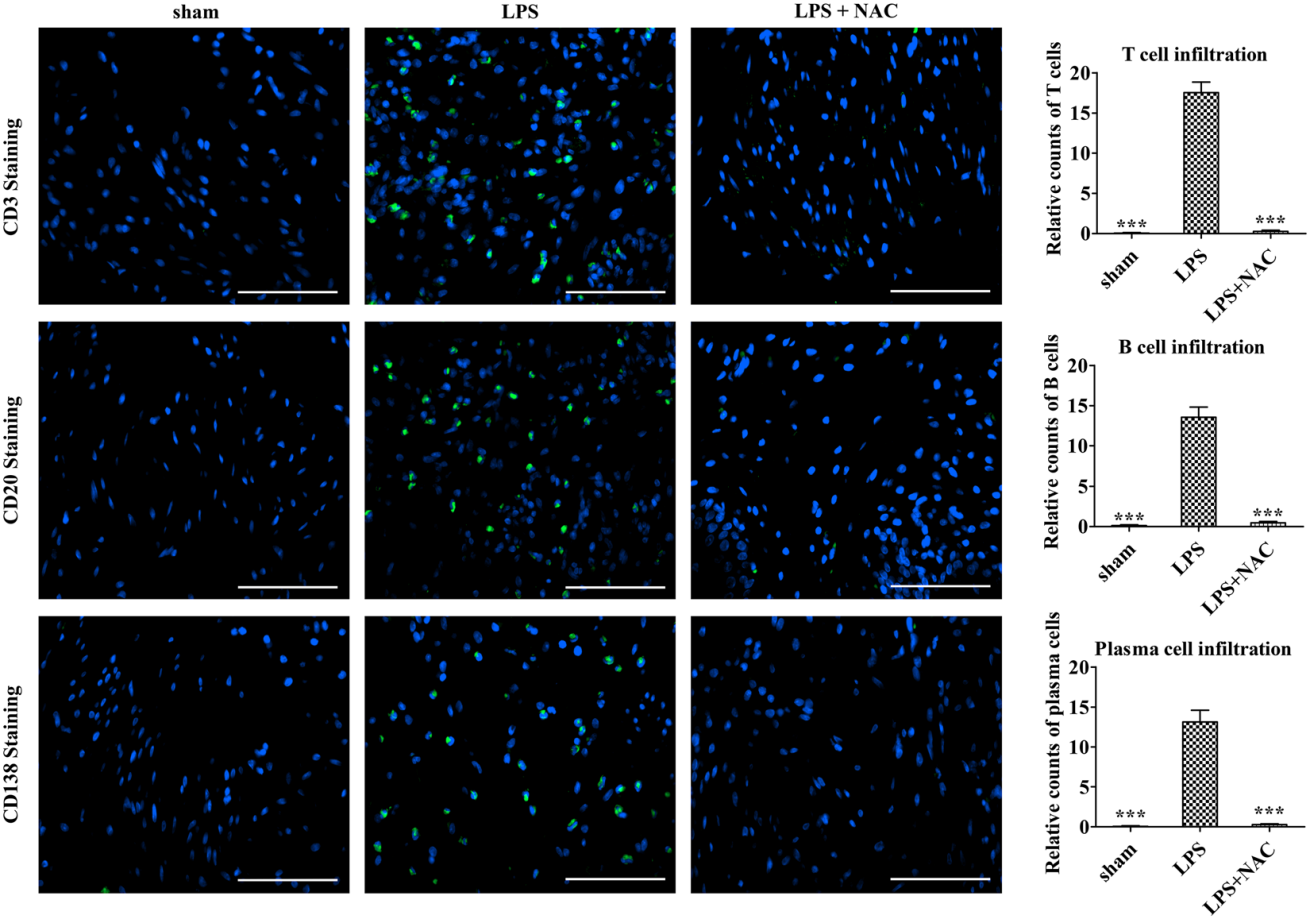
Injection of N-acetylcysteine (NAC) ameliorates histological abnormalities in the bladder of LPS-induced cystitis. Histological analysis of lymphoplasmacytic infiltration in the bladder tissues of the indicated groups. Fluorescent immunohistochemical detection of CD3, CD20 and CD138 protein (green) in the indicated bladder tissues (magnification × 400). Nuclei were stained with 4′,6-diamino-2-phenylindole (blue). Quantitative data of each staining are presented on the right side of the indicated representative pictures. All quantitative data were normalized to those of the sham group and are presented as dot plot with mean ± standard error of the mean (n = 15). *p < 0.05, **p < 0.01, ***p < 0.001 compared with the LPS with the Bonferroni post-test.

We evaluated the beneficial effects of NAC on several histological changes that resulted from LPS instillation. First, double-frequency LPS instillation in the current model exhibited marked increases in urothelial denudation, mast cell infiltration, and apoptosis, which are similar to our previous model^17^. NAC injection effectively reversed those characteristic pathological features of the LPS-treated bladder (Fig. 4a). Next, quantification of CD3-positive T lymphocytes, CD20-positive B lymphocytes, and CD138-positive plasma cells showed that the LPS group contained higher density of lymphoplasmacytic infiltration than the sham group, which indicates that this model is representative of chronic inflammation. On the contrary, LPS-NAC group presented significantly decreased lymphoplasmacytic infiltration compared to the LPS group, indicating that NAC injection poses anti-inflammatory effects (Fig. 5).

**Figure 6:**
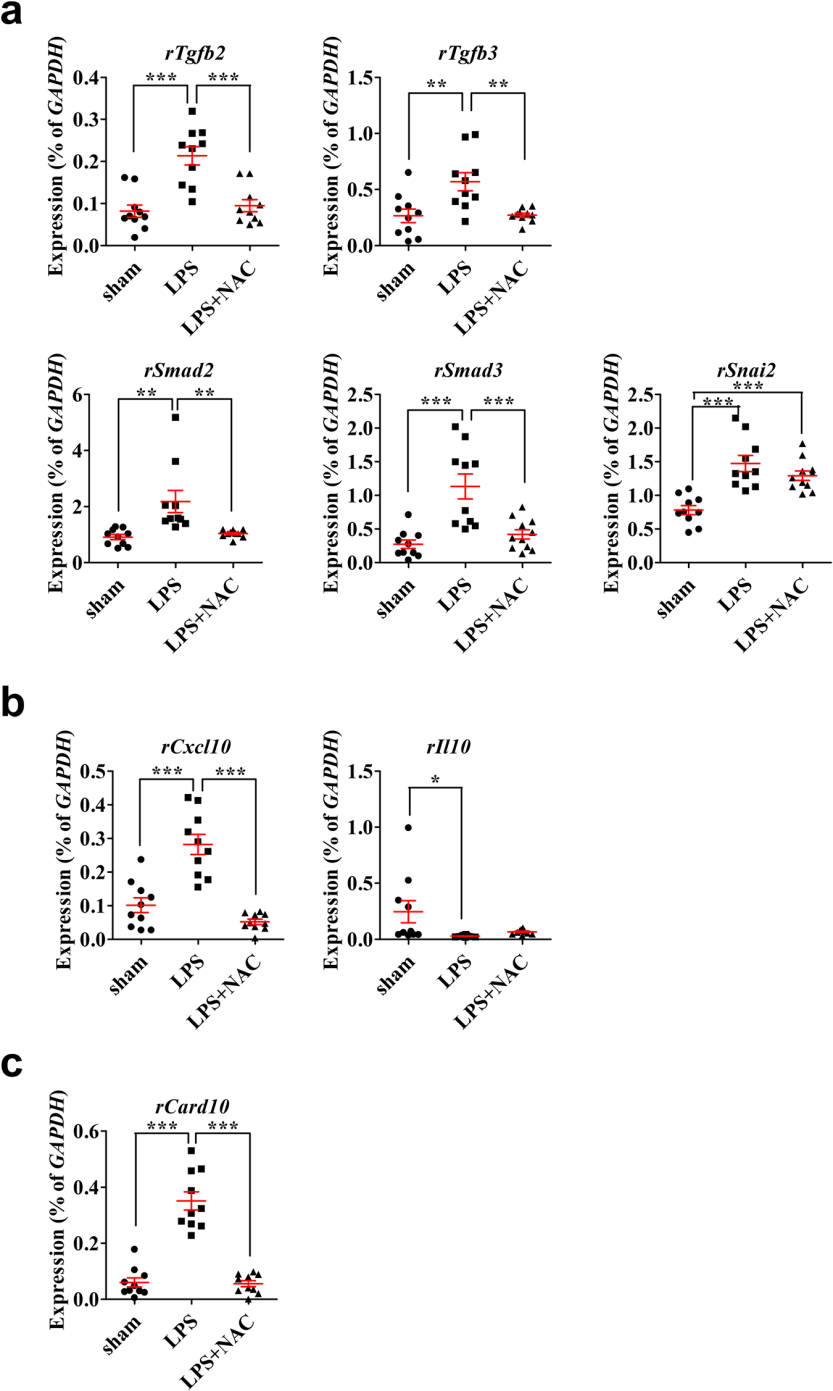
Effect of N-acetylcysteine (NAC) therapy on the expression of genes related to the pathogenesis of LPS-induced cystitis. (**a**–**c**) Real-time quantitative polymerase chain reaction analysis of genes related to tissue fibrosis (**a**), inflammation (**b**), and apoptosis (**c**) in the indicated bladder tissues. Expression is presented as %*Gapdh* and shown as dot plot with mean ± standard error of the mean (n = 10). *p < 0.05, **p < 0.01, ***p < 0.001 compared with the LPS group. ns, nonsignificant.

NAC treatment significantly protected against the fibrotic change and p-Smad2 activation induced in the LPS-treated bladders (Fig. 4b,c). Gene expression analysis showed that a subset of tissue fibrosis-associated genes, (encoding TGF-β2 and TGF-β3 (*Tgfb2* and *Tgfb3*), SMAD family members 2 and 3 (*Smad2* and *Smad3*), and snail family zinc finger 2 (*Snai2*)) was upregulated markedly in the bladders of IC group rats, and the activation of these TGF-β pathway genes except *Snai2* was attenuated by NAC injection (Fig. 6a). As in our previous study with a single dose of LPS instillation^17^, the regimen with double LPS instillation upregulated the expression of proinflammatory chemokine C-X-C motif ligand 10 (*Cxcl10*), but downregulated the expression of the anti-inflammatory cytokine *Il-10* (Fig. 6b). In the LPS group, caspase recruitment domain family member 10 (*Card10*, which performs a role in apoptosis) was upregulated significantly (Fig. 6c). Importantly, NAC treatment effectively prevented the upregulation of *Cxcl10* and *Card10*; however, it had little effect on *Il-10* transcripts levels (Fig. 6b,c).

## Discussion

In the present study, we developed a cystitis rat model with chronic inflammation and prominent fibrosis by increasing the frequency of intravesical LPS instillation. This LPS-induced cystitis rat model exhibited urothelial denudation, mast cell infiltration, and fibrosis histologically and bladder dysfunction which were similar to IC. In addition, intraperitoneal injection of NAC improved inflammation, fibrosis, and voiding parameters in LPS-induced cystitis rats.

During the fibrosis process, endothelial damage activates circulating platelets with exposed collagen and von Willebrand factor in the subendothelial layer^25^. Activated platelets release platelet-derived growth factor, which attracts inflammatory cells, and TGF-β1, which stimulates the extracellular matrix with local fibroblasts^26^. TGF-β is also produced by macrophages that appear in the early stage of the wound-healing process^27^. In this rat model, LPS injury promoted the deposition of fibrotic extracellular matrix proteins and nuclear accumulation of p-Smad2 protein, indicating the activation of TGF-β signaling. Importantly, these fibrotic responses in the bladders of LPS group rats were significantly improved by NAC treatment. According to gene expression analysis, the LPS injury upregulated a subset of genes related to tissue fibrosis, inflammation, and apoptosis, but suppressed the transcription of *Il-10*, an anti-inflammatory cytokine. NAC treatment significantly prevented the activation of the transcription of TGF-β pathway genes including the proinflammatory *Cxcl10* and apoptosis-related *Card10*. Thus, further studies are required to investigate the functional cascade and the interplay of these pathways, as well as to identify which target(s) could explain the therapeutic effects of NAC.

Fibrosis of bladder is well-observed in substance-induced cystitis, such as ketamine and cyclophosphamide, or radiation cystitis. Chronic exposure to irritants and sustained inflammation result in urothelial damage, cellular edema, substitution of smooth muscle to fibroblasts, and deposit of collagen in detrusor^28^. Bladder becomes fibrotic with reduced capacity and compliance. Possible treatment modalities—for example, indwelling urinary catheter, self-catheterization, augmentation cystoplasty or cystectomy with urinary diversion—are either circumventing the problem or highly invasive^29^.

In IC/BPS, clinical symptoms related to fibrosis are relatively uncommon, but histologically intra- and inter-fascicular fibrosis is common in Hunner-type IC^30^. Although debatable, bladder tissue of non-Hunner type IC patients presented predominant fibrosis and mast cell infiltration in our previous study^12^. Patients with different histology require different endoscopic treatment. In patients with Hunner lesions in the bladder (IC), transurethral resection and cauterization of the lesion proved effective in ameliorating symptoms^31^. In patients without Hunner lesions in the bladder (BPS), the most common treatment is hydrodistension of the bladder^31^. During hydrodistension, the bladder is filled with normal saline to the maximum capacity at a fixed pressure that is maintained for a set period of time. However, hydrodistension has a short efficacy duration^32,33^. Currently, treatment modalities for bladder fibrosis (regardless of severity) are limited, and NAC, proven to have therapeutic effects by reducing inflammation and reversing fibrosis, could be a possible breakthrough.

However, clinical application of NAC remains a challenge. The high dose of NAC resulted in dimethylarsinic acid-induced inflammation and cell proliferation, leading to papillary and nodular hyperplasia of the urothelium in the rat model by Takahashi *et al*.^34^ NAC is presumed to enhance oxidative stress and to upregulate extracellular signal regulated kinase (ERK) 1/2 and cyclin D1. Upregulation of ERK signaling is consistent with an early change in urinary carcinogenesis in humans^35^. NAC is a thiol (-SH) compound with the ability to directly scavenge hydroxyl radicals. The interaction of reactive radicals with thiols could generate thiyl radicals, resulting in increased oxidative stress^36^. In a previous study, inhaled NAC promoted the generation of hydroperoxide in chronic obstructive pulmonary disease. Moreover, oral administration of 1.2 and 2.4 g/day of NAC to healthy volunteers resulted in increased blood concentration of glutathione disulfide (oxidized glutathione), showing that NAC acts like a pro-oxidant at these dosages^37^. The establishment of an optimal clinical protocol including dosage and injection route is necessary.

Substance-induced cystitis and radiation cystitis are intractable, and IC/BPS is a heterogeneous disorder with a poorly understood pathophysiology. Although several treatment options are available, they are not reliable, and many patients experience treatment-associated mortality, or persistent or recurrent symptoms, giving rise to the idea that bladder fibrosis cannot be cured. In this study, NAC-based therapy had beneficial effects in repairing voiding function and regenerating the denuded urothelium, as well as in relieving tissue fibrosis and inflammation in the LPS-induced cystitis rat model. Considering the limited treatment approaches for chronic inflammation and detrusor fibrosis, NAC may be a new therapeutic approach.

## Materials and Methods

### Animal models and NAC administration

All animal experiments were approved and done in full compliance with the guidelines of the Institutional Animal Care and Use Committee of the University of Ulsan College of Medicine (IACUC-2016-12-088). For LPS instillation, female 8-week-old Sprague-Dawley rats (OrientBio, Gapyong, Gyeonggi-do, Korea) were administered with protamine sulfate (PS, 10 mg/rat; Sigma-Aldrich, St. Louis, MO, USA) through the urethra by using a 26-gauge angiocatheter in order to denude the urothelium. After 45 min, the bladders were emptied, washed with phosphate-buffered saline solution, and treated with LPS (750 μg/rat, Sigma-Aldrich) for 30 min to induce inflammation. Twice-weekly instillations of PS/LPS following this regimen over a period 5 weeks were used to induce a long-lasting and possibly chronic injury to the urothelium. One week after the final instillation of PS/LPS, 200 mg/kg NAC (Sigma-Aldrich) was administered to a group of PS/LPS-instilled rats through daily intraperitoneal injection for 5 days, followed by a 2-day rest period (LPS + NAC group, n = 10). As a control, the same volume (500 μL) of phosphate-buffered saline without NAC was injected into the sham and LPS groups (n = 10). Immediately after awake cystometry, bladder tissue was harvested for histological analysis. All experimental procedures are described in Fig. 1a.

### Awake and unstrained cystometry

Bladder function was evaluated two weeks after final instillation of PS/LPS in the awake condition with the unrestrained model animals in metabolic cages. Three days before cystometry, intravesical and intra-abdominal pressure were recorded, as described previously^38^. The urethra was approached using a PE-50 catheter (Clay Adams, Parsippany, NJ, USA) with a pressure transducer (Research Grade Blood Pressure Transducer; Harvard Apparatus, Holliston, MA, USA) and microinjection pump (PHD22/2000 pump, Harvard Apparatus). The voiding volumes were recorded via a fluid collector with a force displacement transducer (Research Grade Isometric Transducer, Harvard Apparatus) while the bladder was infused with normal saline at a rate of 0.4 mL/min. The intravesical pressure, intra-abdominal pressure, and voiding volumes were recorded continuously using Acq Knowledge 3.8.1 and the MP150 data acquisition system (Biopac Systems, Goleta, CA, USA) using a sampling rate of 50 Hz. The mean values from three reproducible voiding cycles in individual animals were used for analysis. A nonvoiding contraction was counted when the increments of intravesical pressure exceeded 15 cmH_2_O from baseline without expelled urine. Bladder pressure (BP) was defined as the lowest BP value during filling, micturition pressure (MP) as the maximum BP during the micturition cycle, micturition volume (MV) as the urine volume of expelled urine, and residual volume (RV) as the urine volume remaining after voiding. Bladder capacity (BC) was defined as MV + RV, and micturition interval (MI) as the interval between micturition contractions.

### Histological examinations

Histological analysis of the expression in the bladder tissues rats was performed two weeks after the final instillation of PS/LPS to evaluate epithelial denudation, mast cell infiltration, tissue fibrosis, and apoptosis with cytokeratin immunostaining (Keratin, Pan Ab-1; Thermo Scientific, Foster City, CA, USA), Masson’s trichrome staining (Junsei Chemical, Tokyo, Japan), toluidine blue staining (Toluidine blue-O; Daejung Chemicals & Metals, Seoul, Korea), and terminal deoxynucleotidyl transferase dUTP nick end labeling (TUNEL) staining (Roche, Mannheim, Germany), respectively, as previously described^38,39^. For lymphoplasmacytic infiltration analysis, we used the antibodies CD3 (sc-80668; Santa Cruz Biotechnology, Dalla, TX, USA), CD20 (sc-393894; Santa Cruz Biotechnology, Dallas, TX, USA) and CD138 (ab34164; Abcam, Cambridge, MA, USA) to detect T-lymphocytes, B-lymphocytes, plasma cells, respectively.

In brief, after fixation in 4% paraformaldehyde for 24-h, bladders were each embedded in paraffin, cut on a microtome slices of 3-μm thickness, affixed to slides, and with hematoxylin and eosin (H&E) stained. Fibrosis and mast cell infiltration and were assessed using toluidine blue staining and Masson’s trichrome staining. To measure apoptosis, the bladder was stained with antibodies specific for TUNEL, and the status of TGF-β signaling was examined using immunostaining with phosphorylated Smad2 (p-Smad2; Ser465/467; Cell Signaling Technology). All immunohistological stainings were visualized by Alexa 488-conjugated anti-rabbit antibody (Molecular Probes, Grand Island, NY, USA). The nuclei were counterstained with 4′,6-diamino-2-phenylindole. Quantitative digital image analysis was performed for each slide in seven randomly selected representative areas. To quantify fibrosis and apoptosis, areas positively stained with Masson’s trichrome and TUNEL were evaluated using Image-Pro 5.0 software (Media Cybernetics, Rockville, MD, USA). Mast cells, activation of TGF-β signaling, and lymphoplasmacytes were quantified by means of counting cells, which were respectively stained with toluidine blue, and p-Smad2 and CD antibodies. Three randomly chosen areas from each slide (n = 15) from five independent animals were used to quantify the histological digital image data.

### Gene expression analysis

Using the RNeasy-Mini Kit (Qiagen Inc., Valencia, CA, USA), total RNA from bladder tissues was isolated, and the DNA-free Kit (Applied Biosystems, Foster City, CA, USA) was used to remove genomic DNA. Using Taqman reverse transcription reagents (Applied Biosystems), mRNA (400 ng) was reverse transcribed per manufacturer’s instructions. Quantitative assessment of the target genes’ expression levels was performed using real-time quantitative PCR (RQ-PCR) with a PikoReal™ Real-Time PCR System (Thermo Scientific) with iQ™ SYBR Green PCR Master Mix (Bio-Rad, Hercules, CA, USA), as described previously^39,40^. Gene expression data were quantified using duplicated RQ-PCR assays (n = 10) from five independent animals.

### Statistical analysis

Data were reported as mean ± standard error of the mean and were analyzed using GraphPad Prism 7.0 (GraphPad Software, La Jolla, CA, USA). Differences and significance were verified using 1-way or 2-way ANOVA followed by Bonferroni post-hoc tests. P-values of <0.05 were considered statistically significant.

## Supplementary information

Figure S1
