# Supplementary material for: N-acetylcysteine prevents bladder tissue fibrosis in a lipopolysaccharide-induced cystitis rat model
Source: Sci Rep. 2019 May 31;9:8134. doi: 10.1038/s41598-019-44631-3 (PMC6544636; doi:10.1038/s41598-019-44631-3)
Supplement: Supplementary file 1 — Figure S1 [file 41598_2019_44631_MOESM1_ESM.docx]

**N-acetylcysteine prevents bladder tissue fibrosis in a lipopolysaccharide-induced cystitis rat model**

Chae-Min Ryu^1,2,†^, Jung-Hyun Shin^1†^, Hwan Yeul Yu^1,2^, Hyein Ju^2,3^, Sujin Kim^2,3^, Jisun Lim^2,3^, Jinbeom Heo^2,3^, Seungun Lee^2,3^, Dong-Myung Shin^2,3,*^, Myung-Soo Choo^1,*^

^1^Department of Urology, ^2^Department of Biomedical Sciences, and ^3^Department of Physiology, Asan Medical Center, University of Ulsan College of Medicine, Seoul, Korea

^†^These authors contributed equally to this work.

**Running title: Anti-fibrotic effect of NAC on LPS-IC rats**

**^*^Correspondence:**

Myung-Soo Choo, MD, PhD, Department of Urology, Asan Medical Center, University of Ulsan College of Medicine, 88 Olympic-ro 43-gil, Songpa-gu, Seoul 05505, Korea

Tel.: 82-2-3010-3735; Fax: 82-2-477-8928; E-mail: mschoo@amc.seoul.kr

Dong-Myung Shin, PhD, Department of Biomedical Sciences, Asan Medical Center, University of Ulsan College of Medicine, 88 Olympic-ro 43-gil, Songpa-gu, Seoul 05505, Korea

Tel.: 82-2-3010-2086; Fax: 82-2-3010-8493; E-mail: d0shin03@amc.seoul.kr

**Keywords: N-acetylcysteine, fibrosis, interstitial cystitis, lipopolysaccharide**

**SUPPLEMENTARY FIGURE LEGENDS**

**
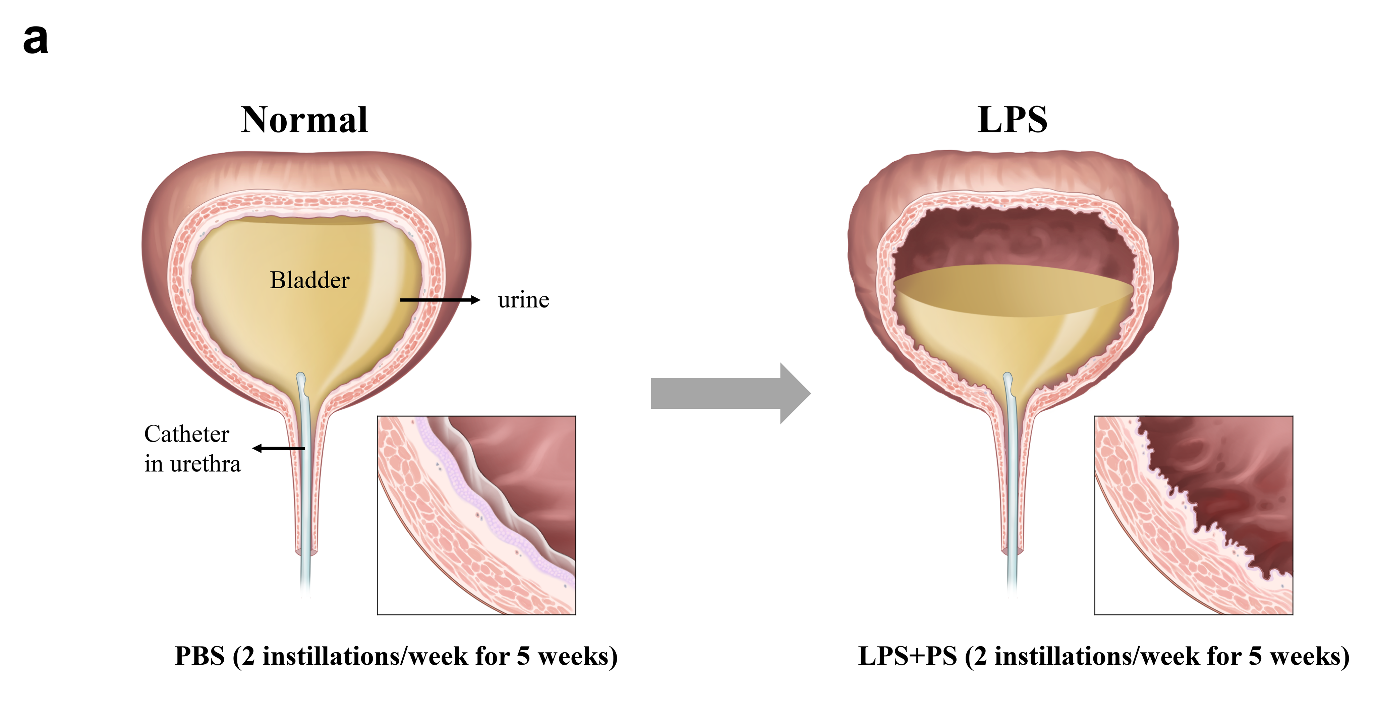
**

**Figure S1. Schematic illustration of experiment.**

Intravesical LPS instillation (twice per week for 5 weeks) resulted in urothelial denudation and fibrotic change in whole bladder compared to sham group (intravesical PBS instillation twice per week for 5 weeks).
